# Interferon β-Mediated Protective Functions of Microglia in Central Nervous System Autoimmunity

**DOI:** 10.3390/ijms20010190

**Published:** 2019-01-07

**Authors:** Stefanie Scheu, Shafaqat Ali, Ritu Mann-Nüttel, Lisa Richter, Volker Arolt, Udo Dannlowski, Tanja Kuhlmann, Luisa Klotz, Judith Alferink

**Affiliations:** 1 Institute of Medical Microbiology and Hospital Hygiene, University of Düsseldorf, 40225 Düsseldorf, Germany; Shafaqat.Ali@med.uni-duesseldorf.de (S.A.); Ritu.Mann@med.uni-duesseldorf.de (R.M.-N.); lisa.richter.14@uni-duesseldorf.de (L.R.); 2 Department of Psychiatry and Psychotherapy, University of Münster, 48149 Münster, Germany; volker.arolt@ukmuenster.de (V.A.); udo.dannlowski@ukmuenster.de (U.D.); judith.alferink@ukmuenster.de (J.A.); 3 Cells in Motion, Cluster of Excellence, University of Münster, 48149 Münster, Germany; 4 Institute of Neuropathology, University Hospital Münster, 48149, Münster, Germany; Tanja.kuhlmann@ukmuenster.de; 5 Department of Neurology, University of Münster, 48149 Münster, Germany; Luisa.klotz@ukmuenster.de

**Keywords:** CNS autoimmunity, experimental autoimmune encephalomyelitis, multiple sclerosis, microglia, type I IFN, IFNβ

## Abstract

Multiple sclerosis (MS) is a chronic inflammatory disease of the central nervous system (CNS) leading to demyelination and axonal damage. It often affects young adults and can lead to neurological disability. Interferon β (IFNβ) preparations represent widely used treatment regimens for patients with relapsing-remitting MS (RRMS) with therapeutic efficacy in reducing disease progression and frequency of acute exacerbations. In mice, IFNβ therapy has been shown to ameliorate experimental autoimmune encephalomyelitis (EAE), an animal model of MS while genetic deletion of IFNβ or its receptor augments clinical severity of disease. However, the complex mechanism of action of IFNβ in CNS autoimmunity has not been fully elucidated. Here, we review our current understanding of the origin, phenotype, and function of microglia and CNS immigrating macrophages in the pathogenesis of MS and EAE. In addition, we highlight the emerging roles of microglia as IFNβ-producing cells and vice versa the impact of IFNβ on microglia in CNS autoimmunity. We finally discuss recent progress in unraveling the underlying molecular mechanisms of IFNβ-mediated effects in EAE.

## 1. Introduction

Multiple sclerosis (MS) is a chronic inflammatory disease of the central nervous system (CNS) characterized by demyelination and neurodegeneration. Neuropathological hallmarks of MS comprise multifocal demyelinated plaques in the CNS composed of inflammatory cells, gliosis, and partial axonal loss [1]. Clinical presentations of MS are characterized by exacerbation and remission of neurological symptoms such as paresthesia, spasticity, urinary dysfunction, and cognitive deficits. Relapsing-remitting MS (RRMS) represents the most common disease course. A minority of about 15% of all patients develops primary-progressive MS (PPMS) characterized by continuous deterioration of neurologic function in the absence of acute exacerbations [2]. Although the etiology of MS remains elusive, it is well established that the immune system is involved in demyelination leading to axonal injury and loss [3,4,5,6].

Interferon (IFN) β represents a well-established treatment strategy used in patients with RRMS [7]. Together with multiple IFNα subtypes (11 in mice and 13 in humans), IFNβ represents the main constituent of the type I IFN family [8,9]. Type I IFNs fulfill key functions in anti-viral immunity and are involved in immune responses against bacteria, parasites, and fungi [10]. On the other hand, dysregulated expression of this cytokine family can lead to the development of inflammatory autoimmune diseases [11]. All type I IFNs signal through a common heterodimeric IFNα receptor (IFNAR) to activate the Janus kinase (JAK)-signal transducer and activator of transcription (STAT) pathway. This leads to the transcription of IFN-stimulated genes (ISGs) [12,13]. ISG-encoded proteins mediate the induction of cell-intrinsic antimicrobial states in infected cells itself and in neighboring cells that limit the spread of infectious agents, particularly viral pathogens. Additionally, ISGs influence innate and adaptive immune responses by promoting antigen presentation, activating T, B, and natural killer (NK) cell responses, and modulating inflammatory cytokine production [14]. Type I IFN production, however, can also have a deleterious effect in chronic infections and thus promote inflammatory disorders and autoimmunity [10,11,15,16]. On a molecular level, type I IFN induction and signaling is therefore tightly controlled by negative feedback regulation, e.g., by the induction of suppressor of cytokine signaling (SOCS) proteins 1 and 3 or ubiquitin specific protease (USP) 18, which compete with STAT proteins and displace JAK1 from binding to the cytoplasmic domains of the IFNAR complex, respectively [12,17,18,19]. Type I IFNs are induced by binding of highly conserved pathogen-associated molecular patterns (PAMPs) but also endogenous agonists associated with tissue damage to membrane bound or cytosolic pattern recognition receptors (PRR) such as Toll-like receptors (TLRs), retinoic acid inducible gene (RIG)-I-like receptors (RLRs), or members of the DEAD-box helicase (DDX) family [20,21,22,23].

Since 1995, various IFNβ preparations have been licensed for the treatment of RRMS in Europe [24]. Therapy with IFNβ has been established as a first-line treatment for RRMS and shown to the reduce relapse rate and delay progression of disability. However, nonresponse rates of up to 49% have been reported [25,26]. Furthermore, a substantial proportion of IFNβ-treated patients develop neutralizing antibodies against IFNβ that limit effectiveness of treatment [27,28]. Adverse side effects such as depression, flu-like symptoms, and lymphocytopenia may also limit IFNβ application [29]. The majority of RRMS patients develop secondary-progressive MS (SPMS), a disease form characterized by a progressive worsening of neurologic function and an increase in disability [30,31,32]. With regard to IFN-β treatment in the management of SPMS, large-cohort studies gave discrepant results [33]. Until now, additional disease modifying and immunomodulatory therapies for RRMS have been licensed. Among those glatirameracetate [34], dimethyl fumarate [35], teriflunomide [36], fingolimod (Sphingosin-1-Phosphat receptor agonist) [37], and cladribine [38], as well as the humanized monoclonal antibodies Natalizumab, that inhibits lymphocyte migration into the CNS by blocking the adhesion molecule very late antigen-4 (VLA-4) [39], Alemtuzumab, that recognizes CD52 on lymphocytes resulting in T and B cell depletion [40,41], and Ocrelizumab, an anti-CD20 antibody specifically depleting B cells, have been approved [42]. Notably, beside its approval for RRMS, Ocrelizumab has been additionally licensed for treating PPMS. Progress in MS therapies have been reviewed in detail before and is therefore not further discussed here [2,43,44,45,46,47].

With the aim to increase efficiency and to reduce side effects of IFNβ therapy, tremendous efforts have been undertaken to understand IFNβ–mediated actions in the pathogenesis of RRMS and have also been studied in the murine model, experimental autoimmune encephalomyelitis (EAE) [48]. EAE reproduces certain clinical and neuropathological features of MS. Neuroinflammation and neurodegeneration in EAE are substantially modulated by microglia, immune competent cells within the CNS, and CNS-infiltrating peripheral macrophages [49]. In CNS autoimmunity, the protective, pleiotropic effects of IFNβ are undisputed, based on findings demonstrating that IFNβ therapy ameliorates EAE. Likewise, induction of endogenous IFNβ production by polyinosinic:polycytidylic acid (poly(I:C)) treatment leads to reduced EAE symptoms in mice while animals deficient for IFNβ or its receptor exhibit increased clinical severity [50,51,52]. The underlying mechanisms mediating the actions of endogenously produced or exogenously applied IFNβ in CNS autoimmunity are still not fully understood [53].

Here we review the current understanding of the impact of microglia and CNS invading macrophages during CNS autoimmunity. We focus on recent advances in the identification of IFNβ-producing cells and IFNβ-mediated molecular pathways in CNS autoimmunity. We finally highlight the cellular targets of this cytokine in EAE.

## 2. Origin and Phenotype of Microglia and Macrophages in the CNS

Microglia were discovered a century ago and are the primary innate immune cells of the CNS [54,55,56]. They orchestrate innate and adaptive immune responses and represent the first line of defense against pathogens invading the CNS. Additional myeloid cells such as perivascular macrophages and macrophages in the choroid plexus and the meninges are present in the CNS at steady state, and blood-derived monocytes and dendritic cells (DCs) invade the CNS under inflammatory conditions [57]. Microglia activation has been implicated in various CNS disorders, neuroinflammation, and neurodegeneration [58,59]. However, under non-inflammatory conditions, microglia also play central roles as effectors of neuronal circuit reorganization and are involved in the development and homeostasis of the brain [54,60,61,62,63].

Microglia and macrophages represent phagocytes of the brain with the capacity to internalize pathogenic particles through various mechanisms such as phagocytosis, pinocytosis, and receptor-mediated endocytosis [64]. Microglia and macrophages have originally been thought to originate from the same myeloid precursors, hematopoietic stem cells in the bone marrow (BM) [65,66]. In the last years, parabiosis and fate-mapping experiments as well as comprehensive transcriptome analyses have fully revised this view. It is now established that microglia entirely arise from a subset of erythromyeloid precursors from the yolk sac (YS), a process that involves the spleen focus forming virus (SFFV) proviral integration oncogene SPI1 encoding the PU-box binding transcription factor (Pu.1) and interferon regulatory factor 8 (Irf8) transcription factor signaling. In analogy to microglia, highly specialized macrophages in several peripheral tissues represent descendants from embryonic progenitors from the YS with the capacity to self-renew in situ. Only to a minor extent, these phagocytes are replaced by circulating monocytes [67,68,69]. Microglial progenitors seed the brain early in embryogenesis and continuously proliferate until the early postnatal phase [70,71]. In the adult, microglia homeostasis is highly dependent on interleukin colony-stimulating factor-1 (CSF-1) receptor signaling. In addition to CSF-1, the stroma-derived cytokine IL-34 represents an alternative ligand for the CSF-1 receptor (CSF-1R). CSF-1R deficient mice lack microglia while deficiency in IL-34 results in a major decrease but not an absolute loss of microglia indicating that CSF-1 may partially compensate for IL-34 under these conditions [72,73,74]. Various experimental models of microglia ablation have defined microglia as long-lived cells in the CNS that self-renew under physiological conditions [75]. The chemokine receptor Cx3Cr1 is highly expressed in microglia and binds neuron-derived fractalkine or Cx3Cl1 [76,77]. Microglia depletion in Cx3Cr1^creER^ mice crossed to a mouse line containing an inducible gene-cassette coding for the diphtheria toxin receptor (iDTR) demonstrated that a minor subset of ablated microglia is replaced by CNS-invading monocytes [78]. In addition, the pharmacological blockade of CSF-1 receptor signaling resulted in ablation of microglia in mice and a rise of novel parenchymal microglia in the CNS after the treatment has been finished [79]. Recent studies performing large scale single-cell RNA-sequencing further discovered that in addition to microglia, perivascular, meningeal, and choroid plexus macrophages, but not monocytes, stem from the YS and have the capacity of self-renewal [80].

Microglia express PRRs such as TLRs to sense PAMPs. Additionally, host-derived ligands such as heat shock proteins, S100 proteins, high mobility group box 1, or DNA-binding proteins when released into the extracellular space [81] are recognized as damage-associated molecular patterns (DAMPs) and trigger microglial activation in the absence of infection [82]. Activation of microglia results in a change of their morphology into an amoeboid shape and an enhanced migratory capacity [59]. Various factors and receptors have been shown to be involved in the transition from a resting to an activated microglia, such as Runt-related transcription factor 1 (Runx1), Irf8, Pu.1, as well as triggering receptor also expressed on myeloid cells-2 (TREM2), Cx3Cr1, and CD200R [59,61,70,83]. As discussed below in detail, the concept of M1 and M2 activation states of macrophages has also been adopted for microglia and linked to distinct functional properties [84,85]. In the next chapter, we will therefore highlight current knowledge on the polarization and function of microglia and macrophages in CNS autoimmunity.

## 3. Functions of Microglia and Macrophages in CNS Autoimmunity and Models of Toxic Demyelination

### 3.1. Microglia Activation and Polarization in Neuroinflammation

Microglia and macrophages play major roles in the pathogenesis of MS and in murine models of this disease. More than twenty years ago, accumulation of macrophage-like cells, as determined by immunohistochemistry, has been documented in early MS lesions [86]. Later studies documented the presence of CCR1^+^ and CCR5^+^ expressing monocytes as well as CCR1^−^ CCR5^−^ putative microglial cells in early demyelinating stages [87]. Based on findings that microglial and macrophage activation is associated with signs of demyelination in MS samples, it was proposed that both, resident microglia and blood-derived macrophages may contribute to demyelination and neuronal damage in MS [88,89].

Interestingly, the histological classification system of MS lesions has been updated recently based on a comprehensive quantification of their macrophage and microglia content and demyelinating activity. MS lesions have been redefined into “active”, “mixed active/inactive” and “inactive” lesions. Active and mixed active/inactive lesions can further be subdivided into lesions with ongoing myelin destruction and post-demyelinating lesions based on the presence of cytoplasmic myelin degradation products in macrophages/microglia [90]. TMEM119 is a marker that might allow differentiation between microglia and blood derived monocytes in human tissue sections [91,92]. In early stages of active lesions approximately 43% of Iba1 positive cells also express TMEM119 [93]. Interestingly, mixed active/inactive lesions characterized by a rim of phagocytes are the dominating lesion type in progressive MS and therefore might be the driver of disease progression [94]. Also, in this rim a significant number of TMEM119 positive cells was found, further suggesting that microglial cells contribute to disease progression in MS [93].

Positron emission tomography (PET) imaging studies using various ligands, among those ligands with affinity to the microglial marker translocator protein 18 kDa (TSPO), have been conducted to determine microglial activation in MS in vivo. TSPO is expressed on the outer mitochondrial membrane and in activated microglia [95,96]. However, increased TSPO expression in PET imaging studies of MS patients has also been linked to density but not activation of these cells [97]. Enhanced microglial activation has been documented in Magnetic Resonance Imaging (MRI)–detected inflammatory lesions in MS patients with active disease, while the association between the level of TSPO binding, clinical disability, and the effect of MS therapeutics on microglial activation are still discussed [98]. With regard to M1/M2-like microglia, radioligands targeting the purinergic P2X7 receptor that is expressed on M1 microglia have already been studied in animal models and in human studies. However, multiple splice variants and genetic polymorphisms of the *P2X7* gene are known to potentially affect its biological function and binding potential of tracers and may thus limit its usability as PET imaging target. Another interesting candidate as future PET imaging target represents the purinergic receptor, P2Y12, that is upregulated in alternatively activated human M2 microglia [99]. However, final proof of its usability as a PET imaging target in MS is still missing, see [96,98].

Under inflammatory conditions, i.e., in the presence of the TLR4 ligand lipopolysaccharide (LPS) or interferon γ (IFNγ), M1 microglia are induced that express enhanced surface levels of MHC class II and costimulatory CD40 and CD86 molecules, macrophage migration inhibitory factor (MIF) receptor CD74, and chemokine receptor CCR7. M1 microglia produce pro-inflammatory cytokines (e.g., IL-1β, IL-6, IL-18, tumor necrosis factor (TNF), and chemokines, enhance NO synthase activity and thus promote inflammation and oligodendrocyte damage [100,101,102]. In this sense, microglia activation orchestrates the recruitment of leucocytes to inflammatory sites and executes local effector functions aimed at controlling infections, while aberrant or prolonged microglia activation may aggravate neuroinflammation and neurodegeneration [103]. Furthermore, microglial dysfunction and release of pro-inflammatory cytokines may alter glutamate homeostasis and thus evoke glutamate excitotoxicity leading to neuronal death [104,105]. In MS, the imbalance in glutamate homeostasis has been reported to contribute to oligodendroglial and axonal pathology underscoring the involvement of glutamate excitotoxicity as an important mechanism in autoimmune demyelination [106,107]. Microglia activation further plays an essential role in inflammation-mediated neurodegeneration during acute as well as chronic stages of MS via triggering production of reactive oxygen and nitrogen species that promote progressive neurodegeneration in MS [108]. However, also anti-inflammatory phenotypes of M2 microglia have been reported, that are characterized by expression of e.g., mannose receptor CD206, scavenger receptors, and anti-inflammatory IL-10. M2 microglia have been associated with resolution of inflammatory responses and repair mechanisms in the CNS [109,110,111,112,113]. It has been suggested that classically activated M1 microglia may play a role in promoting inflammation and damage of oligodendrocytes, while M2 microglia may promote repair mechanisms and remyelination [84,110,114,115,116]. However, there is still controversy on this topic, since microglia may also display intermediate phenotypes [117,118,119]. In MS, an “intermediate activation status” of microglia cells has been reported based on findings demonstrating that they co-express M1- and M2-like markers such as CD40 and mannose receptor in inflammatory MS lesions [118]. This subject has been reviewed in detail before [120].

### 3.2. Impact of Microglia and Macrophages in Myelin Oligodendrocyte Glycoprotein-EAE

EAE is a CD4^+^ T-helper (Th)-cell-mediated autoimmune disease in rodents induced by immunization with CNS-specific antigens such as myelin oligodendrocyte glycoprotein (MOG). After induction of IL-17 producing CD4^+^ Th cells (Th17) via presentation of MOG by peripheral antigen presenting cells (APCs), Th17 cells re-encounter their cognate antigen on local and CNS-invading APCs after CNS recruitment [101,121,122,123]. This induces a second wave of leucocyte recruitment to the CNS that results in demyelination and axonal degeneration. Macrophages and DCs accumulate in the inflammatory infiltrates with higher cell numbers correlating with increased disease severity [124,125,126,127]. Microglia activation in EAE was studied in CD11b-HSVTK transgenic mice that express the cell suicide gene herpes simplex virus thymidine kinase (HSVTK) in macrophages and microglia. The authors generated BM chimeric mice by reconstitution of irradiated CD11b-HSVTK mice with wildtype (WT) BM to prevent hematopoietic toxicity after ganciclovir administration and found that ganciclovir treatment inhibited the development of EAE [128]. These data indicated that microglial activation plays a key role in the pathogenesis of EAE. However, in these studies the contribution of CNS macrophages and also CD11b-expressing DCs could not be fully excluded.

Microglia and peripheral monocytes exhibit overlapping markers. There is an ongoing debate whether a unique marker or a combinatorial set of markers may specifically identify microglia versus CNS immigrating macrophages in neuroinflammation [129]. As discussed above, recent finding highlight TMEM119 as a specific microglial marker in human tissue sections [91,92,93,94]. The development of strategies to distinguish infiltrating macrophages and resident microglia in neuroinflammation is thus a rapidly growing research field and has been reviewed in more detail before [70,130,131]. In the following we highlight selected studies on this topic. Durafourt and co-workers demonstrated that M2 polarizing conditions lead to the induction of a unique cell surface marker profile in microglia from the adult human CNS as compared to monocyte-derived or circulating macrophages. While CD209 (DC-SIGN) has been detected on M2 microglia, additional receptors such as the low affinity IgE receptor CD23, the scavenger receptor CD163, and the mannose receptor CD206 have been found exclusively on M2 macrophages. Importantly, microglia were superior in myelin phagocytosis when compared to macrophages independent of the respective polarization condition [132]. To technically discriminate and to study the specific functional role of CNS infiltrating versus resident CNS myeloid cells in the demyelinating process, elaborate techniques such as parabiosis, generation of BM chimeras, and/or inducible gene targeting models were utilized. For example, in Ccr2^rfp/+^::Cx3cr1^gfp/+^ mice, CX3CR1-expressing microglia can be identified via green fluorescence protein (GFP), while red fluorescent protein (RFP) expression marks peripheral CCR2^+^ monocytes. This approach allows to study each cell type specifically using histological strategies and fluorescence cytometric expression profiling. At MOG-EAE onset, monocyte-derived macrophages (MDMs) in Ccr2^rfp/+^::Cx3cr1^gfp/+^ mice have been found to invade nodes of Ranvier and initiate demyelination. MDMs further show distinct morphological characteristics when compared to microglia-derived macrophages (MiDMs), the latter exhibiting larger size and multiple primary processes. Analysis of gene expression profiles revealed that MDMs express more genes encoding effector functions when compared to MiDMs suggesting that both cell types may exert distinct roles in the pathogenesis of EAE [133,134]. Another study points toward distinct features of microglia versus blood-derived myeloid cells in EAE. Wlodarzyk and coworkers studied three myeloid cell subsets in the CNS of EAE-induced mice, namely CD11c^+^ and CD11c^−^ microglia, and CD11c^+^ blood-derived cells. They demonstrated that CD11c^+^ and CD11c^−^ microglia and infiltrating CD11c^+^ peripheral myeloid cells represent distinct populations. Depending on CD11c expression, microglia show distinct expression patterns of Arg-1, YM1, iNOS, IL-10, and IGF-1, while CD11c^+^ as well as CD11c^−^ microglia subsets were capable to produce IFNβ in contrast to CD11c^+^ blood-derived cells [135]. A recent study using an inducible and microglia specific deletion of the aryl hydrocarbon receptor (AhR) demonstrated that microglia may also regulate the pathogenic activities of astrocytes in EAE development by controlling transforming growth factor α (TGFα) and vascular endothelial growth factor (VEGF)-B production by these cells. Via secretion of TGFα microglia limit pathogenic astrocyte actions and development of EAE, whereas microglial-derived VEGF-B enhanced FLT-1 signaling in astrocytes and aggravates EAE [136].

### 3.3. Impact of Microglia and Macrophages in Cuprizone and Lysolecithin-Induced Demyelination

Microglia activation has further been implicated in cuprizone-induced neuropathology. In this model, demyelination in the corpus callosum is induced by a diet with the toxic copper chelate cuprizone and remyelination after termination of the diet [137,138]. In contrast to EAE, demyelination in this model takes place in the absence of peripheral immune inflammation. Cuprizone rather induces apoptosis of oligodendrocytes leading to degeneration of myelin sheaths. Conversely, remyelination is initiated already during cuprizone treatment by intra-CNS recruitment of oligodendrocyte progenitor cells (OPCs) that differentiate into myelin-producing oligodendrocytes after toxin-withdrawal [139]. Activated microglia accumulate in mice after cuprizone treatment primarily in the corpus callosum but have also been found in the cortex and hippocampus [140,141]. Comparative analysis of microglia gene expression from the corpus callosum of cuprizone-fed mice during demyelination versus remyelination points toward the presence of a typical remyelination-supportive phenotype of microglia. This phenotype is characterized by a repertoire of cytokines and chemokines that promote recruitment and differentiation of OPCs. Interestingly, this phenotype was maintained during de- and remyelination indicating that on the one side microglia mediate phagocytosis of myelin debris during demyelination and on the other side promote OPC functions during remyelination [117]. Voss and colleagues demonstrated that demyelination was associated with upregulated expression of MHC class II molecules, TNF, as well as phagocytic receptors most prominently TREM-2b pointing towards a multifunctional microglial phenotype [119]. Of note, TNF- and MHC class II-deficient mice show defective or delayed remyelination, respectively, underscoring the importance of both molecules in this model [142,143]. Infiltrating monocytes have been attributed a lesser functional role as they represent only a minor fraction, 5% or less, of the CD11b^+^ cells in the corpus callosum after cuprizone diet [119,144,145]. Myelin debris, that represents an in vitro inhibitor of OPC differentiation, has also been shown to impair CNS remyelination by inhibiting OPC differentiation [146,147]. For further details on the role of microglia in remyelination following review is recommended [137].

A mouse model of focal demyelination is induced by intrathecal or intraspinal microinjection of lysolecithin [148,149]. After lysolecithin treatment, demyelinated areas harbor infiltrating macrophages/microglia, activated astrocytes, axonal injury, and proliferating OPCs. During demyelination, Mac3^+^ activated microglia have been identified in the lesions and shown to decline in numbers during remyelination [148]. It is important to bear in mind that many earlier studies did not distinguish between macrophages and resident microglia due to usage of overlapping markers. Nevertheless, several findings clearly point toward a functional involvement of macrophages/microglia in the inflammatory response in de- and remyelination. For example, SJL/J mice show reduced macrophages/microglia numbers associated with enhanced remyelination in the lesion site after treatment with immunosuppressive corticosteroids [150]. In addition, progesterone treatment of male C57BL/6 mice increases circulating corticosteroid levels to those in female pregnant mice, enhanced anti-inflammatory and remyelinating effects by microglial/macrophages, and reduced lysolecithin-induced damage [151]. Early studies further used silica exposure of rodents to inhibit macrophage responses [152]. This treatment in rats has been shown to counteract demyelination further underscoring detrimental actions of macrophages/microglia. However, no influence on remyelination after lysolecithin-treatment was observed under these conditions [153]. It has further been proposed that M1 microglia mediate the inflammatory CNS response during lysolecithin-induced demyelination that results in oligodendrocyte damage, while M2 microglia promote repair mechanisms [154]. Interestingly, the same group demonstrated that OPC recruitment was delayed when macrophages were depleted by clodronate-liposomes in lysolecithin-treated mice [154,155]. Accordingly, parabiosis experiments have recently demonstrated that specifically young macrophages enhance remyelination via myelin debris clearance [156]. Also, minocycline-mediated inhibition of microglia activation impairs OPC responses and remyelination in this model [157]. Focal demyelination by stereotactic injection of lysolecithin into the mouse corpus callosum has further been shown to induce a switch of the M1- to M2- phenotype in immigrating macrophages and microglia at the start of remyelination associated with enhanced phagocytotic capacities. M2 depletion in cerebellar slice cultures further inhibited oligodendrocyte differentiation, a precondition for remyelination, suggesting that M2 cell polarization is required for remyelination [158].

In the following, we summarize research on the cell types responsible for IFNβ production and its impact on microglia effector functions in EAE including references to MS where applicable.

## 4. Microglia as Producers of Endogenous IFNβ in CNS Autoimmunity

IFNβ was discovered as an antiviral factor produced by fibroblasts after viral infections [159]. While IFNβ was originally thought to be produced by virtually all cells of the body, it is now assumed that various types of professional APCs are the major source in immune activation. Following stimulation of PRRs such as TLRs and RIG-like helicases, plasmacytoid and conventional DCs produce IFNβ alongside different IFNα subtypes [12,160,161]. In mouse models for viral infections of the CNS, brain resident cell types such as microglia but also astrocytes and, to a lesser extent, infected neurons have been shown to produce IFNβ [162,163,164]. In Aicardi-Goutières Syndrome (AGS), a rare autoinflammatory neurodevelopmental disorder, initially astrocytes were reported to produce predominantly IFNα based on post mortem histological sections [165]. However, in the AGS mouse model of deficiency in the exonuclease Trex1, cells of neuroectodermal origin, such as astrocytes, did not induce a type I IFN-response. In contrast, Trex1-deficient microglia showed cell intrinsic activation of IFN-stimulated genes indicating type I IFN production by these cells [166].

Also in MS, IFNβ production was detected in cells defined as macrophages and astrocytes based on their morphology in active CNS lesions of patients [167]. In contrast, in the animal model of EAE, based on morphological studies it was suggested that IFNβ may be produced by ramified microglia or infiltrating cells [51]. Using an IFNβ/yellow fluorescence protein (YFP) fluorescence reporter mouse model, IFNβ expression was characterized in situ in the CNS after induction of MOG-EAE. This IFNβ/YFP reporter mouse model defined activated microglia as the prominent IFNβ-producing cells in the CNS that closely associate with myelin debris in inflamed lesions at the peak of EAE [168]. Immigrating myeloid cells may contribute to the IFNβ production as IFNβ/YFP reporter activity was also observed in fewer cells that coexpressed the chemokine receptor CCR2 in neuroinflammation [168]. In this context, CCR2 represents a selective marker for infiltrating macrophages and has here been used to distinguish these cells from resident microglia [134,169,170]. Thus, at the peak of EAE, IFNβ expression has been detected exclusively in CNS myeloid cells in IFNβ/YFP reporter mice and elevated concentrations of IFNβ have been found in the CNS of WT animals [50,168]. Early after MOG-immunization, however, IFNβ production was rather found in peripheral lymphoid organs. This initial expression of IFNβ is induced in plasmacytoid DCs by immuno-adjuvant containing PAMPs thus exacerbating the clinical course of EAE, presumably via contributing to the priming of encephalitogenic T cells [171]. Indeed, type I IFNs produced in the periphery can reach the CNS during neuroinflammation when the blood brain barrier (BBB) integrity is compromised [172].

The exact nature of the agent activating IFNβ production in microglia in neuroinflammation in the context of EAE is still not known. An interesting hypothesis has been put forward based on findings that in the absence of the ubiquitin specific protease USP18, which downregulates type I IFN receptor signaling, microglia in the CNS are hyperactivated already at steady state conditions [173]. Thus it can be assumed that baseline levels of type I IFNs may be present in the CNS even under homeostatic conditions. Indeed, measurable type I IFN protein levels were detected in the CNS of untreated mice in the above cited study by Prinz et al. [50]. This is kept in check by suppressive modulators such as USP18. It has been speculated that oligodendrocytes or OPCs represent the cellular source of low level of type I IFN in the CNS [17]. Alternatively, it is also possible that microglia activate themselves in an auto- and paracrine way to produce type I IFN in response to myelin debris present in the surrounding.

Another study examined the therapeutic role of type I IFN produced within the CNS during EAE after direct administration of poly(I:C) into the cerebrospinal fluid (CSF) [174]. This led to a quick mobilization of IFNβ producing myeloid cells from the periphery into the CNS. Transient IFNβ expression by myeloid cells was detected in meninges and choroid plexus but also microglia exhibited increased IFNβ expression as shown by an IFNβ luciferase reporter mouse model [175]. Thus, these studies point toward distinct expression patterns of IFNβ during homeostasis and EAE possibly linked to distinct functions. In the following chapter IFNβ-mediated beneficial effects on microglial cells during neuroinflammation in CNS autoimmunity will be discussed.

## 5. Protective Effects of IFNβ on Microglia as Effector Cells in CNS Autoimmunity and Toxic Models of Demyelination

Various mechanisms underlying the protective effects of IFNβ in MS and EAE have been suggested [176]. Additionally, IFNβ has been attributed with immunomodulatory functions in models of toxic demyelination. However, for the latter the contribution of microglia produced IFNβ remains to be defined. In the following chapter we will give an update on these more general modes of action of IFNβ before focusing on its microglia-specific effects.

### 5.1. IFNβ-Mediated Immunomodulatory Actions in EAE and Toxic Models of Demyelination

IFNβ-mediated effects in CNS autoimmunity include a generalized reduction of inflammation and tissue damage via induction of apoptosis in peripheral immune cells [177], and downregulation of matrix metalloproteinases and the adhesion molecule very late antigen-4 (VLA-4) [178,179]. In addition, IFNβ has been shown to induce down-modulation of T cell responses via reduced surface expression of MHC class II and costimulatory molecules (CD40) on APCs combined with upregulation of the inhibitory PD-L1 and PD-L2 ligands [180,181,182]. Recently, it has been demonstrated that IFNβ-deficiency in DCs leads to upregulation of CCR7 as well as increased DC migration to the CNS during EAE [183]. In EAE as well as in MS, conventional FoxP3^+^ T regulatory cells have been suggested to play an important role in controlling the autoimmune process [184,185,186]. Unchanged numbers of these FoxP3^+^ T regulatory cells, however, have been reported in EAE in the absence of IFNβ [187]. Instead, type I IFN receptor mediated signaling in neurons is essential to generate FoxA1^+^ T regulatory cells via upregulation of PDL1 in a PI3K-Ak-dependent way [187,188]. These recently defined regulatory FoxA1^+^ T cells act suppressive in EAE in mice and were further found increased in responders versus non-responders to IFNβ therapy [187].

IFNβ-deficient mice have been shown to exhibit an earlier onset of clinical disease and enhanced neuroinflammation during MOG-EAE when compared to WT controls. These animals exhibited increased numbers of Th17 and CD11b^+^ cells in the CNS, while IFNβ treatment of WT animals induced a delayed onset and milder disease. These findings point towards suppressive effects of IFNβ on Th17 cells [189]. Additional studies in MS patients demonstrated that IFNβ resulted in decreased IL-23 mRNA levels and elevated expression of IL-23 were found in untreated MS patients, a cytokine that promotes Th17 cells [190]. Furthermore, IFNβ treatment of DCs from untreated MS patients down-regulated release of IL-23, but enhanced IL-27 gene expression in these cells, a cytokine that may inhibit Th17 differentiation [190,191].

As already highlighted above, endogenous IFNβ production can also be induced by peripheral application of poly(I:C) or other ligands of TLR3 and intracytoplasmatic receptors of the family of RIG-like helicases and several of these ligands were shown to suppress EAE symptoms [52,192,193]. Therapeutic inhibition of established EAE after administration of poly(I:C) into the CSF correlated temporally with type I IFN expression in the CNS and was IFNAR1 dependent. Accordingly, intrathecal poly(I:C) injection into IFNAR1-deficient mice did not result in downregulation of clinical severity and neuroinflammation of EAE. In the same study, levels of IL-10 expression in the CNS were significantly increased in response to poly(I:C) [174]. Production of anti-inflammatory IL-10 by T cells or macrophages has been shown before to be promoted by type I IFN and to mediate beneficial effects via suppression of pathogenic Th17 cells in autoimmune inflammation [194,195,196]. In contrast, in the aforementioned study no effect was reported on expression levels of Th1 and Th17 associated cytokines IFNγ and IL-17, presumably because Th cell differentiation has fully been established at the timepoint of therapeutic intervention. Intrathecally induced type I IFNs instead stimulated glial chemokine CXCL10 production within the CNS [174]. Type I IFN produced within the CNS, therefore, mediates endogenous neuroprotection.

Several studies investigated the role of IFNβ during remyelination after toxic CNS demyelination, albeit with controversial results. An early study demonstrated that IFNβ treatment inhibits in vitro differentiation, but not proliferation, of OPCs. This detrimental effect on OPC differentiation, a prerequisite for remyelination, was mediated indirectly via cocultured astrocytes and microglia [197]. In a follow-up in vivo study, this lab investigated the functional role of IFNβ in de- and remyelination using cuprizone-mediated toxic CNS demyelination. The authors demonstrated that IFNβ-deficient mice showed early on an enhanced spontaneous remyelination associated with increased numbers of OPCs in demyelinated lesions. After six weeks, however, the amount of remyelination did no longer differ compared to WT controls. These findings suggest that IFNβ inhibits recruitment and proliferation of OPCs and thus prevents early remyelination in the absence of inflammation-driven demyelination [198]. In the same model during acute demyelination, upregulated expression of IFNβ and IFN-dependent genes IRF7, ISG15, and UBP43 indicated that IFNAR signaling is induced. However, IFNAR1-deficient mice did not exhibit changes in demyelination, astrocytosis and microgliosis, motor skills, and myelin recovery, indicating that endogenous IFNAR signaling did not alter myelin damage and repair [199].

Only few studies up to now have investigated the effect of IFNβ in the model of lysolecithin-induced demyelination in mice. IFNβ-deficient mice show diminished astrocytic and microglia responses at the start of the remyelination phase and higher numbers of OPCs and enhanced remyelination within the lesion when compared to WT controls. Six weeks after lysolecithin-treatment, however, the amount of remyelination was equivalent in both groups of mice. The authors concluded that IFNβ drives recruitment and proliferation of OPCs possibly already during demyelination, thus promoting early remyelination [198].

### 5.2. IFNβ Mediated Effects on Microglia in CNS Autoimmunity

The specific effects that IFNβ exerts on microglia have also been the topic of various studies in vitro and in vivo in murine disease models. Here it is important to discriminate between the impact of exogenously administered IFNβ from IFNβ that is endogenously produced.

**Figure 1:**
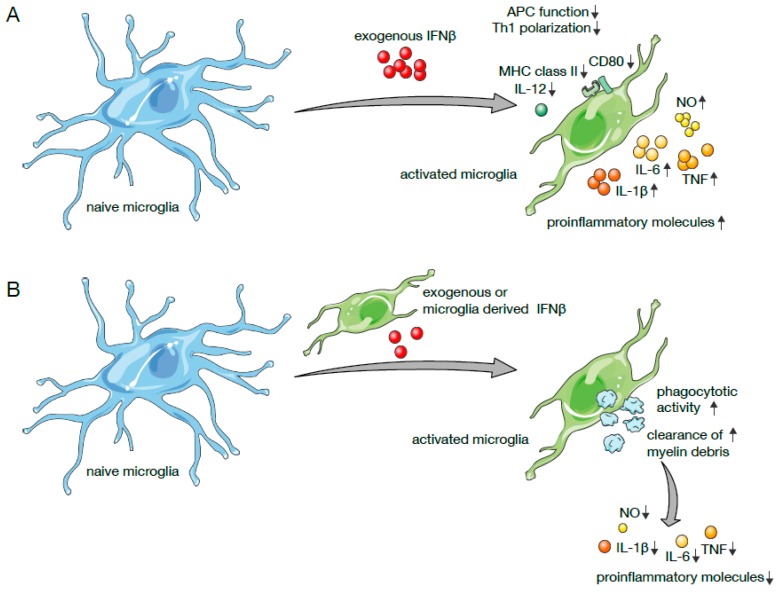
Molecular effects mediated by IFNβ on microglia cells: (**A**) Exogenously added IFNβ downregulates antigen presenting functions such as expression of MHC class II and costimulatory molecules and Th1 polarization while production of proinflammatory cytokines and molecules (TNF, IL-1β, IL-6, NO) is increased. (**B**) IFNβ produced by microglia or added exogenously activates phagocytic activity and increases removal of myelin debris by microglia suggested to cause downregulation of pro-inflammatory factors. Small black arrows indicate up or down regulation of expression of the respective molecules by microglia, grey arrows indicate causative effects. The panel was created using Servier Medical Art according to Creative Commons Attribution 3.0 Unported License (https://creativecommons.org/licenses/by/3.0/). Changes were made to the original cartoons.

Exogenously added IFNβ affects antigen presenting functions such as the expression of MHC class II molecules and costimulatory molecules of in vitro cultured murine microglia as well as the production of proinflammatory cytokines and polarization towards M1 phenotype in these cells (Figure 1A) [200]. Recently it has also been shown that IFNβ produced by microglia or added exogenously activates phagocytic activity in microglia leading to increased removal of myelin debris (Figure 1B) [168,173]. In order to efficiently manage myelin debris removal, microglia that produce IFNβ are themselves strategically positioned within active CNS lesions in EAE in vivo [168]. Also, in lysolecithin-demyelinated cerebellar organotypic slice cultures (OSCs), specifically areas surrounding IFNβ-producing microglia, contained lower amounts of myelin debris [168]. This, in turn, was dependent on type I IFN receptor mediated signaling since OSCs from IFNAR1 deficient mice did not show this effect. These observations are in accordance with data from microglia deficient in the TIR domain containing adapter inducing interferon beta (TRIF) that are less efficient in clearing axonal debris. This directly implicates that the IFNβ activating signal transduction pathway downstream of TLR3 and TLR4 is involved in controlling phagocytic activity in microglia because these cells exhibited an increased threshold for activation of ISGs [201]. Of note, IFNβ producing microglia might activate the orchestration of phagocytosis of myelin debris not only in neighboring microglia but also in phagocytes immigrating into the CNS in the course of autoimmunity. Also, base line type I IFN expression may serve to activate microglia sufficiently to degrade myelin debris that constantly accrues in the aging process of myelin sheaths [202,203].

Clearance of myelin debris has been suggested to ameliorate disease via downregulation of transcription of pro-inflammatory factors like TNF, IL-1β, or inducible nitric oxide synthase (iNOS) and thus reducing IFNγ-mediated neuroinflammation in MS as well as EAE (Figure 1B) [204]. Further, overexpression of the phagocytosis triggering receptor TREM2 reduced the severity of clinical symptoms in EAE [205]. IFNβ-induced reduction of myelin debris load might indeed be a critical repair mechanism in the resolution of lesions and in axonal regeneration, a continuous process in the dynamic equilibrium of inflammation-induced damage and wound healing in MS and EAE. Myelin debris in this sense impairs remyelination by at least two possible mechanisms: it inhibits differentiation and recruitment of OPCs [146] and contains growth inhibitory molecules such as Nogo-A downregulating axonal re-growth [206,207]. Furthermore, protective effects can be assumed via the induction of a regulatory type of microglia resembling M2 macrophages [208]. This substantially amends the functional spectrum of microglia, a cell type attributed so far mostly roles in the inflammatory response during infections and CNS autoimmunity [209].

The role of microglia as target cells of endogenous IFNβ executing the protective mechanisms in CNS autoimmunity is in accordance with studies using mice with a cre-mediated cell type-specific deletion of the type I IFN receptor [50]. The authors showed that type I IFN receptor expression on myeloid cells, that also include microglia, has a nonredundant protective effect in EAE as signified by increased disease severity in LysM-cre driven type I IFN receptor deletion. In contrast, this receptor is dispensable for the suppression of EAE on T and B cells and neuroectodermal cells of the CNS [50]. Interestingly, when type I IFN expression was therapeutically induced by activation of cytoplasmic RIG-like helicases with RNA ligands, LysM-cre driven type I IFN receptor deletion on myeloid cells had no impact on the course of disease. In this experimental setting increased type I IFN amounts act on DCs to mediate protective functions, as indicated by using CD11c-cre mice to delete IFNAR expression [192]. These studies revealed important differences in the mode of action between endogenously produced type I IFN and therapeutically induced type I IFN: While endogenous type I IFN exerts its protective effect via microglia, and possibly to a lesser extent infiltrating monocytes, therapeutically induced type I IFN critically relies on DCs as target cells.

## 6. Conclusions

In this review we discussed novel insights into the in vivo expression pattern and function of IFNβ during CNS autoimmunity and models of toxic demyelination and our current understanding of the role of microglia in this process. In vivo, IFNβ reporter mouse models and more conventional mRNA and protein expression analyses have ultimately proven that microglia represent major IFNβ producers during EAE. IFNβ mediated pleiotropic effects involve modulation of pro-inflammatory responses, cell survival, expression of matrix metalloproteinase, and chemokine receptors as well as down-modulation of T cell responses via ameliorating antigen presenting functions. Type I IFN receptor mediated signaling in neurons is further required for induction of FoxA1^+^ T regulatory cells known to suppress immunopathologies. Importantly, non-redundant protective functions of IFNβ in neuroinflammation are mediated by microglia themselves such as the removal of myelin debris in active lesions. Consequently, IFNβ-producing microglia may constitute novel cellular targets to combat detrimental inflammatory responses in CNS autoimmunity. The exact extent to which microglial IFNα/β contributes to IFNAR1-dependent alleviation of EAE remains to be determined and the question how increasing knowledge on CNS-innate IFN-driven regulatory programs could be translated into potential therapies for MS is still unanswered. However, accessing CNS-innate IFNβ-driven regulatory programs represents an attractive therapeutic option in MS and other neurological diseases.

## Abbreviations

AGS	Aicardi-Goutières Syndrome
AhR	aryl hydrocarbon receptor
APC	antigen presenting cells
BBB	blood brain barrier
BM	bone marrow
CNS	central nervous system
CSF	cerebrospinal fluid
CSF-1	colony-stimulating factor-1
DAMP	damage-associated molecular pattern
DC	dendritic cell
DDX	DEAD-box helicase
EAE	experimental autoimmune encephalomyelitis
GFP	green fluorescence protein
HSVTK	herpes simplex virus thymidine kinase
iDTR	inducible diphtheria toxin receptor
IFN	interferon
IFNAR	interferon α receptor
iNOS	inducible nitric oxide synthase
IRF	interferon regulatory factor
ISG	interferon-stimulated gene
JAK	Janus kinase
LPS	lipopolysaccharide
MIF	macrophage migration inhibitory factor
MDM	Monocyte-derived macrophages
MiDM	Microglia-derived macrophages
MOG	myelin oligodendrocyte glycoprotein
MRI	magnetic resonance imaging
MS	multiple sclerosis
NK cell	natural killer cell
NMO	neuromyelitis optica
OPC	oligodendrocyte progenitor cells
OSC	organotypic slice cultures
PAMP	pathogen-associated molecular pattern
PET	positron emission tomography
poly(I:C)	polyinosinic:polycytidylic acid
PPMS	primary-progressive multiple sclerosis
PRR	pattern recognition receptor
RFP	red fluorescence protein
RLH	Retinoic acid inducible gene (RIG)-I like helicase
RRMS	relapsing-remitting multiple sclerosis
Runx1	Runt-related transcription factor 1
SOCS	suppressor of cytokine signaling
STAT	signal transducer and activator of transcription
TGFα	transforming growth factor α
Th cell	T helper cell
TLR	toll-like receptor
TNF	tumor necrosis factor
TREM2	triggering receptor also expressed on myeloid cells-2
TRIF	TIR domain containing adapter inducing interferon beta
TSPO	translocator protein
USP	ubiquitin specific protease
VEGF	vascular endothelial growth factor
VLA-4	very late antigen-4
WT	wildtype
YFP	yellow fluorescence protein
YS	yolk sac
